# Novel Variants of Angiotensin Converting Enzyme-2 of Shorter Molecular Size to Target the Kidney Renin Angiotensin System

**DOI:** 10.3390/biom9120886

**Published:** 2019-12-17

**Authors:** Jan Wysocki, Arndt Schulze, Daniel Batlle

**Affiliations:** 1 Department of Medicine, Division of Nephrology and Hypertension, Feinberg School of Medicine, Northwestern University, Chicago, IL 60611-3008, USA; 2 Department of Medicine, Charité-Universitätsmedizin, D-10117 Berlin, Germany

**Keywords:** ACE2, Renin Angiotensin System, Acute Kidney Injury, Angiotensin II, Angiotensin (1-7)

## Abstract

ACE2 is a monocarboxypeptidase which generates Angiotensin (1–7) from Angiotensin II (1–8). Attempts to target the kidney Renin Angiotensin System using native ACE2 to treat kidney disease are hampered by its large molecular size, 100 kDa, which precludes its glomerular filtration and subsequent tubular uptake. Here, we show that both urine and kidney lysates are capable of digesting native ACE2 into shorter proteins of ~60–75 kDa and then demonstrate that they are enzymatically very active. We then truncated the native ACE2 by design from the C-terminus to generate two short recombinant (r)ACE2 variants (1-605 and 1-619AA). These two truncates have a molecular size of ~70 kDa, as expected from the amino acid sequence and as shown by Western blot. ACE2 enzyme activity, measured using a specific substrate, was higher than that of the native rACE2 (1-740 AA). When infused to mice with genetic ACE2 deficiency, a single i.v. injection of 1-619 resulted in detectable ACE2 activity in urine, whereas infusion of the native ACE2 did not. Moreover, ACE2 activity was recovered in harvested kidneys from ACE2-deficient mice infused with 1-619, but not in controls (23.1 ± 4.3 RFU/µg creatinine/h and 1.96 ± 0.73 RFU/µg protein/hr, respectively). In addition, the kidneys of ACE2-null mice infused with 1-619 studied ex vivo formed more Ang (1–7) from exogenous Ang II than those infused with vehicle (AUC 8555 ± 1933 vs. 3439 ± 753 ng/mL, respectively, *p* < 0.05) further demonstrating the functional effect of increasing kidney ACE2 activity after the infusion of our short ACE2 1-619 variant. We conclude that our novel short recombinant ACE2 variants undergo glomerular filtration, which is associated with kidney uptake of enzymatically active proteins that can enhance the formation of Ang (1–7) from Ang II. These small ACE2 variants may offer a potentially useful approach to target kidney RAS overactivity to combat kidney injury.

## 1. Introduction

Angiotensin Converting Enzyme-2 (ACE2) is a monocarboxypeptidase that degrades several substrates, of which Ang II is most relevant because of the extensive biologic effects of this peptide and the attendant formation of Ang (1–7), which has actions generally opposite to those of Ang II [1,2,3,4,5,6,7,8,9]. ACE2 protein in its full-length form is an 805 amino-acid (AA) type-I transmembrane protein (110–120 kDa) that contains an extracellular domain (AA 1–740), a transmembrane region (AA 741–768), and an intracellular tail (769–805) [10,11]. The extracellular part of ACE2 (1-740 AA), here referred as “native” ACE2, contains the catalytic domain and is fully catalytically active. In our previous studies, the administration of native rACE2 (1-740 AA) resulted in a marked increase in circulating ACE2 activity, but neither kidney nor urinary ACE2 activity increased [6,12,13].

The lack of increase in urinary ACE2 activity after injection of native rACE2 was attributed to the lack of glomerular filtration of native rACE2, consistent with its large molecular size of 100–110 kDa [12,13]. While markedly increased circulatory levels of ACE2 activity after native rACE2 administration are capable of effectively lowering blood pressure in models of Ang II-induced hypertension [6,7,14] or renin overexpression in the circulation [15], their use is not suitable for treatment of other forms of kidney disease. For instance, in a diabetic model with local kidney but not systemic RAS over-activity [13], long-term augmentation of circulating ACE2 activity was not sufficient to alter glomerular pathology, GFR or albuminuria [13]. These observations provide the rationale for the development of shorter forms of ACE2 with a molecular size that render them filtrable by the kidney and, therefore, potentially useful therapeutically to amplify ACE2 activity in forms of kidney disease with an overactive local RAS in the kidney [13,16].

ACE2 is a tissue enzyme with low levels of activity in plasma [13,17]. In urine, enzymatic ACE2 activity is substantial [12,18,19]. Two distinctive ACE2-specific bands have been reported in mice and human urine [18,19,20,21]. One ACE2-immunoreactive band, at about 100–110 kD, corresponds to the expected molecular weight of native ACE2 at 110 kD [12]. This 100–110 kD ACE2-immunoreactive band is likely the result of shedding from the kidney apical tubular membrane, where ACE2 protein is abundantly expressed [22,23,24]. The other ACE2-imunoractive band detectable in mouse urine is of a lower size (~75 kD) and its origin and whether or not it is active remains unclear [12]. Based on these observations, we hypothesized that ACE2 variants of a shorter molecular size could be generated and still retain enzymatic activity. Accordingly, we sought to develop short ACE2 variants small enough, so that they could be filtered and subsequently re-absorbed by the kidney tubules. The kidney uptake of these small ACE2 variants, in turn, would foster Ang (1–7) formation from Ang II, thereby providing a novel and direct approach to target the kidney RAS, which is overactive in some forms of kidney disease, including acute kidney injury.

## 2. Methods

### 2.1. Degradation of Native rACE2 in Urine and Kidney Lysates from ACE2KO Mice

Kidney cortex total protein fractions were extracted as previously described [23,24]. Urines (500 µL; each containing 1–5 mg total protein) and kidney cortex lysates (each containing 1–2.5 mg total protein) from ACE2-deficient mice (C57BL/6 genetic background) were mixed with 50 mM HEPES, pH 7.4, NaCl (150 mM), Triton X-100 (0.5%), ZnCl_2_ (12.5uM end-concentration) and incubated with 1 µg of native mouse mrACE2 (10 nM) at 37 °C constantly shaking at 800 rpm for up to 48 h. At specified time points of incubation, a 50 µL aliquot was taken and mixed with an equal volume of PBS buffer containing EDTA-free inhibitor cocktail (Roche, Basel, Switzerland) and immediately frozen. Each of the aliquots was then used for measuring ACE2 activity using Mca-APK-Dnp substrate and to detect ACE2 protein on Western blot. For Western blot, proteins were separated by SDS-PAGE, and transferred to nitrocellulose membranes. The membranes were blocked in non-fat dry milk (5–7% wt/vol) solubilized in Tris-buffered saline solution containing 0.1% Tween 20. The nitrocellulose membranes were incubated with primary anti-ACE2 antibody [AF3437 (R&D Systems, Minneapolis, MN, USA) or Ab38888 (Abcam, Cambridge, MA, USA)] and horseradish peroxidase-conjugated secondary antibody (Santa Cruz Biotechnology). The specificity of the ACE2 antibodies was confirmed by the absence of any bands in Western blot using ACE2KO samples, as previously reported [12]. Bands were visualized using chemiluminescence system (Super Signal Pico, Pierce, Rockford, IL, USA).

### 2.2. Design of Short ACE2 Variants

To generate short ACE2 fragments that exhibit ACE2 activity, a series of *ace2* DNA constructs of varying lengths were generated through truncation from the C terminus (Supplementary Materials Figure S1). The cDNA of short *ace2* was generated by PCR amplification using as a template the cDNA of the intact soluble mouse *ace2* (coding amino acids 1-740). To gradually shorten ACE2, we used specific primers that determine the length of the shorter ACE2 cDNA to be amplified and have compatibility with the expression vector restriction sites (pcDNA, Invitrogen, Carlsbad, CA, USA). The absence of mutations in the amplified cDNA was verified by sequencing. The plasmids with the inserted cDNAs of the short ACE2 variants were then expressed by transient transfection in HEK293 cells. ACE2 activity was measured in the conditioned culture medium using the fluorometric substrate Mca-APK-Dnp [7] (Supplementary Materials Figure S1). The native rACE2 that contained the full extracellular domain (1-740 AA) was used as a positive control. As a negative control, conditioned media from mock-transfected cells was used. In addition, Western blot using a polyclonal antibody raised against the entire extracellular domain of ACE2 (R&D Systems, AF3437) was used to detect the transgenes and confirm the molecular size of the over-expressed small ACE2 variants.

### 2.3. Production and Purification of Selected Short ACE2 Variants

Two short and enzymatically active ACE2 variants were selected for large scale production and purification. From stably transfected HEK cells, single clones were selected and expanded to 220 mL flasks. Conditioned serum-free medium from a clone of stably transfected HEK 293 cells that overexpressed the two short ACE2 variants was subjected to anion exchange Q-column on AKTA chromatography system in 25 mM Tris-HCl, pH 8.0 and eluted by applying increasing concentration of NaCl. Eluted fractions were screened for ACE2 activity was using Mca-APK-Dnp substrate. Fractions containing ACE2 activity were applied to SDS-PAGE, transferred to PVDF membrane and stained with Brilliant Blue to assess protein purity (Supplementary Materials Figure S2, showing ACE2-1-619 as an example).

### 2.4. Enzyme Activity

To compare the enzymatic activities of highly purified, enzymatically active short ACE2 proteins, we tested their ability to cleave its main natural substrate, Ang II (1–8), to form Ang (1–7). This was measured by an assay measuring the release of the C-terminal amino acid, phenylalanine, which is formed as a byproduct in the cleavage of Ang II (1-8) to Ang (1-7) [25]. The relative enzymatic potency of the short rACE2 fragments was determined by comparison with equivalent molar amounts of the intact rACE2 (740 AA long), which was used as a benchmark. For assessment of activity levels, the Michaelis–Menten model was used to derive the parameters of catalytic kinetics such as Km and Kcat [15].

### 2.5. Acute Blood Pressure Response Ising Ang II-Induced Hypertension Mouse Model

To study the effect of small rACE2 variants on Ang II-induced hypertension, we injected 10–20 week old male C57bl/6 mice i.p. with either vehicle (PBS), mouse rACE2 1-619 or mouse rACE2 1-605 (both 1 µg/g of body weight). After 1 h, mice were anesthetized with an i.p. injection of ketamine (150 mg/kg of body weight). Mice were then placed on a temperature-controlled platform for 10 min immediately after anesthesia was induced. Systolic BP was monitored noninvasively every 30 s for a period of 20 min, as previously described [6,7]. After five minutes of baseline SBP recording, acute hypertension in anesthetized mice was induced with an i.p. bolus injection of Ang II (0.2 mg/kg of body weight), and the SBP was monitored in a consecutive mode, at the same 30 s intervals throughout the remaining 15 min time period.

### 2.6. Recovery of Urinary ACE2 after Recombinant ACE2 Infusion to ACE2-Deficient mice and the Effect of Blocking Tubular Reabsorption with L-lysine

Immediately after the ACE2-deficient mice [26,27] voided urine (baseline urine collection), purified mouse recombinant ACE2 proteins were administered as a single i.v. bolus injection at a dose of 1.0 µg/g body weight. In order to determine the impact of ACE2 protein reabsorption in the proximal tubule, lysine, a blocker of proximal tubular reabsorption [28,29] was also administered (0.4 mg/g body weight) as a single i.p. injection. The timeline of the experiment was as follows: mice were weighted, followed by collection of baseline urine levels, and, within 5–10 min after voiding urine, mice were administered mrACE2 proteins in a single i.v. injection (0.2 mL/mouse, 1 µg/g BW). Then, immediately after i.v. injection, to collect urine, mice were placed for 2 h in urine collection cages with access to water and food. Afterwards, L-lysine was injected i.p. Urine was collected again within 2 h after the subsequent i.p. injection of the tubular reabsorption blocker, L-lysine (0.4 mg/g body weight).

### 2.7. Pharmacokinetics Analysis

The pharmacokinetic profiles of mouse rACE2 1-619 and 1-605 were assessed in Balb/c mice as compared to those of native mouse rACE2 1-740. The mice received a single i.v. or i.p. injection of each purified small rACE2 variant at a dose of 1 µg/g body weight. Blood samples were collected by tail bleeding either before or at a number of indicated time points after injection. Blood samples collected in heparinized capillaries were left undisturbed on ice, and plasma was isolated by centrifugation at 1850 g for 10 min at 4 °C. Mca-APK(Dnp) substrate (Bachem, Bubendorf, BL, Switzerland), PA) was used to measure ACE2 enzyme activity in plasma. The half-life (t_1/2_), area under the plasma activity time curve and mean residence time of rACE2 species were calculated [15] using Prism 8 software (GraphPad, La Jolla, CA, USA).

### 2.8. Statistical Analyses

For comparison of two independent groups, a two-tailed t-test was used if data was normally distributed. The normality of the distribution was assessed using Shapiro-Wilk test. For not normally distributed data, a Mann–Whitney test was used. For comparison of more than two independent groups, one-way ANOVA was employed, followed by Tukey’s multiple comparisons test. For comparisons of Ang (1–7) formation and SBP over time, 2-way Anova was used. A *p*-value < 0.05 was considered statistically significant. Results are presented as mean ± SE.

## 3. Results

### 3.1. Degradation of Native rACE2 to Shorter Enzymatically Active ACE2 Truncates

In mouse urine, two ACE2-immunoreactive bands are present [12]. In fresh kidney lysates, however, only a 100–110 kD ACE2 band was found to be consistent with the molecular size of native mouse ACE2 [12]. It was unclear whether the lower 75 kD band was a product of native ACE2 degradation or a distinct protein. We therefore decided to examine the possibility that the 75 kD band could be a proteolytic digestion product of the 110 kD ACE2 band. To study the hypothesis of ACE2 proteolysis, urines and kidneys from ACE2-deficient mice were spiked with native mrACE2 that we had generated, over-expressed and purified. This native recombinant mouse (mr)ACE2 has a molecular size of 100–110 kD (740 amino acid long) and contains a poly-His tag on its C-terminus [7]. Samples from ACE2 knockout mice were used to eliminate any interference arising from endogenous ACE2.

**Figure 1:**
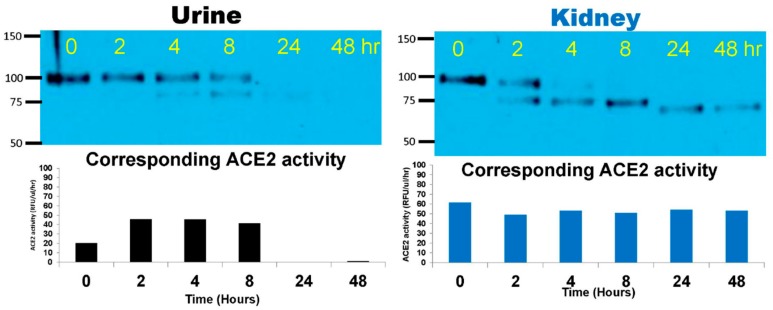
Native mouse recombinant (mr)ACE2 (100–110 kD) was spiked into ACEKO urine (**Left**) or kidney cortex lysate (10 nM mrACE2/~1 mg total protein of the lysate) from one ACE2KO mouse (**Right**) and incubated at 37 °C for 48 h. Spiked native mrACE2 samples at 0, 2, 4, 8, 24 and 48 h of incubation were subsequently probed in Western blot. **Left Panel.** Western blot image of ACE2KO urine spiked with native mrACE2 shows gradual weakening of the 100–110 kD native mrACE2 band and the appearance of smaller 75 kD ACE2 immunoreactive band for up to 8 h. At 24 and 48 h, no ACE2 immunoreactive bands and no ACE2 activity are detectable anymore. **Right Panel.** Western blot image shows the disappearance of the 100–110 kD mrACE2 band and, first the appearance of a smaller 75 kD ACE2 immunoreactive band, and then a ~60–70 kD band in kidney lysates from ACE2KO mice. In the lower panel, ACE2 activity is depicted showing similar enzyme activities of the 75 and ~60 kD bands versus the original 110 kD mrACE2 band throughout the incubation period; a single time-course experiment for kidney and for urine is shown.

After spiking with the native 100–110 kD, mrACE2, ACE2KO urines and kidneys were incubated for up to 48 h at 37 °C to enable proteolysis of the native mrACE2 by urinary and kidney proteases, respectively. Small samples of the reaction mixture were taken for analysis at different time points within the 48 h incubation time. In ACE2KO [23,27] kidney lysates, the extended incubation resulted not only in the formation of a 75 kD band, but also a shorter ~60–70 kD ACE2 immunoreactive band that had significant ACE2 activity (Figure 1). The spiking of native mrACE2 into urine from ACE2 KO resulted similarly in the appearance of a 75 kD band and a gradual fading of the native 100–110 kD mrACE2. In kidney lysate, at 24 and 48 h of incubation, there was a band and no activity, which was not seen in the ACE2KO urine.

**Figure 2:**
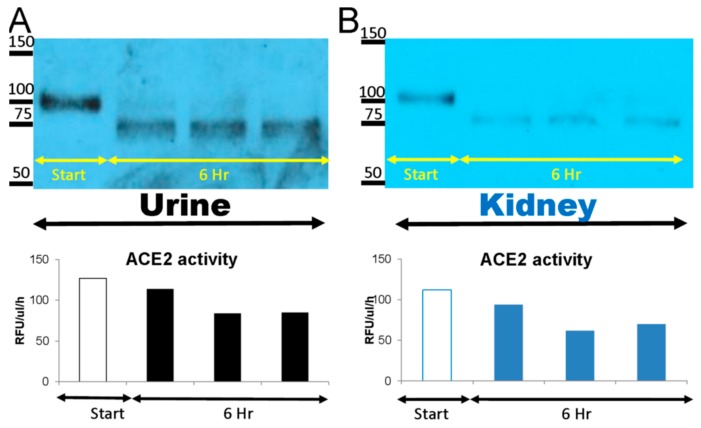
Native mouse recombinant (mr)ACE2 1-740 AA (100–110kD) was spiked into urine (10 nM mrACE2/~0.5 mL urine) (**A**) and kidney cortex lysates (10 nM mrACE2/~1 mg total protein of the lysate) from three ACE2KO mice (**B**) and incubated at 37 °C for 6 h. Spiked mrACE2 samples at 0 h (Start) and at 6 h of incubation were subsequently probed in Western blot using an anti-ACE2 antibody. Western blot image shows the disappearance of the 100–110 kD mrACE2 band and the appearance of a smaller 75 kD ACE2 immunoreactive band at 6 h in urine and kidney lysates from ACE2KO mice. In the lower panel, ACE2 activity is depicted showing the enzyme activity of the 75 kD band and the spiked original 100–110 kD native mrACE2 band; urines and kidney lysates from three independent degradation experiments.

To confirm the consistency of the time course degradation studies just described, three different ACE2KO urines and three different kidney lysates were then incubated with the native 100–110 kD mrACE2 at 37 °C. The six hour timepoint was chosen for the robustness of the bands found in urine and kidney lysates. Consistent with data shown in Figure 1, the 100–110 kD native mrACE2 protein was converted in ACE2KO urines and kidney lysates into a band of an apparent molecular size of 75 kD (Figure 2). This 75 kD ACE2 immunoreactive band appeared to exhibit high specific enzyme activity, similar to that of the original 100–110 kD rACE2 band that has been used for spiking (Figure 2). This suggests that kidney and urine contain proteases capable of digesting native ACE2 into an enzymatically active truncate of ~75 kD.

**Figure 3:**
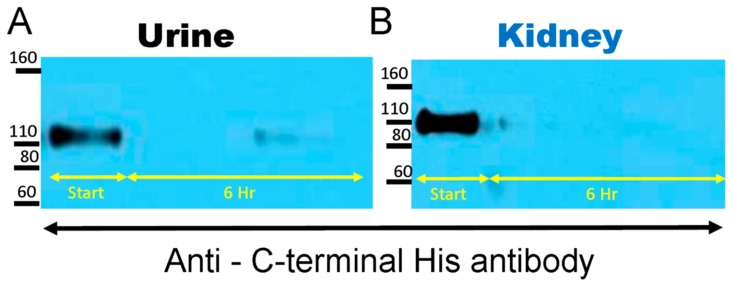
The native mrACE2 spiked into urine (**A**) and kidney (**B**) samples from ACE2KO mice had a c-terminal 10-His tag. The Western blot membrane shown in Figure 2 was re-probed using an anti-His antibody. Western blot image shows disappearance of the 100–110 kD mrACE2 band and no appearance of a smaller 75 kD ACE2 immunoreactive band in urine and kidney lysates from ACE2KO mice, suggesting the proteolysis of native mrACE2 from its c-terminal end; urines and kidney lysates from three independent degradation experiments.

The native mrACE2 that was used for spiking contains a C-terminal 10-His tag. Re-probing of the membranes with anti-His antibody revealed a band of the size of the original native mrACE2 at 100–110 kDa but failed to detect the lower band at ~75 kD (Figure 3). While N-terminal cleavage of native ACE2 cannot be disproved, this finding clearly shows that proteolysis by mouse urine and kidneys takes place at the C-terminal site.

### 3.2. Design of Small ACE2 Variants and Demonstration of Their Enzyme Activity and Molecular Size

Based on the above findings, we then generated C-terminally truncated recombinant mouse ACE2 proteins 1-522, 1-605 and 1-619 (see Methods and Supplementary Materials Figure S1). The molecular size and sequences of three short ACE2 variants, 1-522, 1-605 and 1-619, are provided in the supplement (Supplementary Figure S3). The enzyme activity of those variants was evaluated in conditioned medium and compared to the over-expressed native rACE2 1-740, which was used as a benchmark.

**Figure 4:**
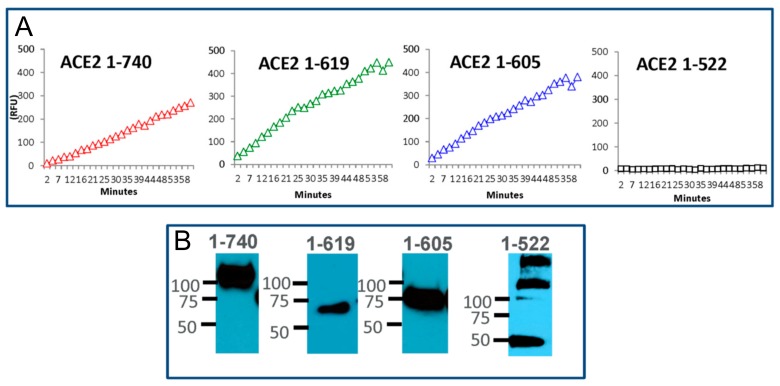
Enzyme activity and molecular size of shorter recombinant ACE2 protein variants. Relative activity of mouse small recombinant ACE2variants and ACE2 protein detection in conditioned media collected 40–60 h after transfection. (**A**) The cleavage rate of the synthetic fluorogenic substrate Mca-AKP(Dnp) over one hour (shown in relative fluorescence units—RFU) for native rACE2 and small ACE2 variants. Similar to native soluble mrACE2 1-740, mrACE2 variants, 1-619- and 1-605- showed enzymatic activity, respectively, while 1-522 did not exhibit any ACE2 activity. (**B)** Presence and estimated molecule size were confirmed by Western Blot with antibody against ACE2 ectodomain.

Enzyme activity in the conditioned culture media, from HEK cells transiently transfected with the ACE2 constructs generated through truncation of the C terminus (1-619 AA and 1-605 AA), was at least as high as in the media collected from cells overexpressing the native rACE2 1-740, as judged by the kinetic slope (Figure 4). In contrast, transfection with a shorter ACE2 fragment (1-522 AA) did not show any enzyme activity (Figure 4A). The two enzymatically active short ACE2 variants (619 and 605) appeared as a single band at the apparent molecular weight of ~70 kDa, as expected from the amino acid sequence deducted using the Expasy Informatics tool (Figure 4B and Figure S3). The enzymatically inactive variant 1-522 appeared, in addition to the expected size of ~50–60 kD, in oligomerized forms of 2–3 bands of higher molecular weight (Figure 4B). This suggests that the minimal length requirement on the C-terminal end for murine ACE2 to be enzymatically active is contained between AA 522 and 605.

### 3.3. Enzyme Activities and Kinetic Constants of Purified Short Recombinant ACE2 Variants Using Angiotensin II as a Substrate

**Figure 5:**
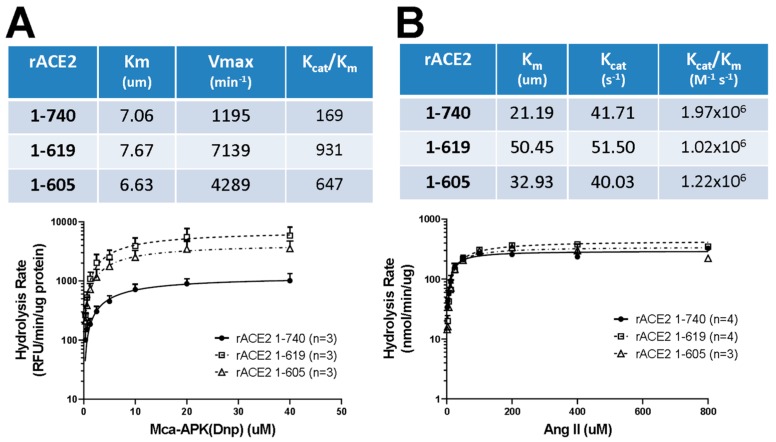
Summary of ACE2-activity assessment of highly purified mouse recombinant ACE2 1-619 and ACE2 1-605 compared to native rACE2 1-740 with a synthetic fluorogenic substrate Mca-APK(Dnp) (**A**) and with the natural substrate, angiotensin II (**B**). A higher catalytic activity (Kcat/Km) was found by cleavage of Mca-APK(Dnp) for rACE2 1-619 and rACE2 1-605 than for native rACE2 1-740 (**Panel A**). The catalytic efficiency to convert Angiotensin II to Angiotensin (1–7) (Kcat/Km) was similar for native rACE2 1-740 and for the small rACE2 variants 1-619 and 1-605 (**Panel B**). For assessment of activity levels, a Michaelis–Menten model was used to derive the parameters of catalytic kinetics such as Km and Kcat. The values were calculated from three to four independent experiments.

Clonal HEK293 cell lines stably expressing the two ACE2 variants (1-605 and 1-619) were established, and the recombinant proteins were purified from the culture medium by strong anion-exchange chromatography, as shown in Supplementary Figure S1A [see details in Methods]. Purified native rACE2 1-740 [7] was used as a control to compare the enzymatic activities of the two short ACE2 variants. The catalytic efficiency of the two short ACE2 variants (Kcat/Km) to cleave the synthetic fluorogenic ACE2 substrate, Mca-APK-(Dnp) [7], was higher as compared to native rACE2. In fact, for the rACE2 1-619, it was four-fold and for rACE2 1-605 three-fold higher as compared to enzymatic activity of the native rACE2 1-740 (Figure 5A). Using Ang II, the natural substrate of ACE2, catalytic efficiencies [K_cat_/K_m_ (M^−1^ s^−1^)] were found to be not different for native rACE2 1-740 (1.97 × 10^6^) and for the two shorter proteins rACE2 1-619 (1.02 × 10^6^) and rACE2 1-605 (1.22 × 10^6^) (Figure 5B) (*n* = 3–4 experiments for each protein).

### 3.4. Pharmacokinetics

**Figure 6:**
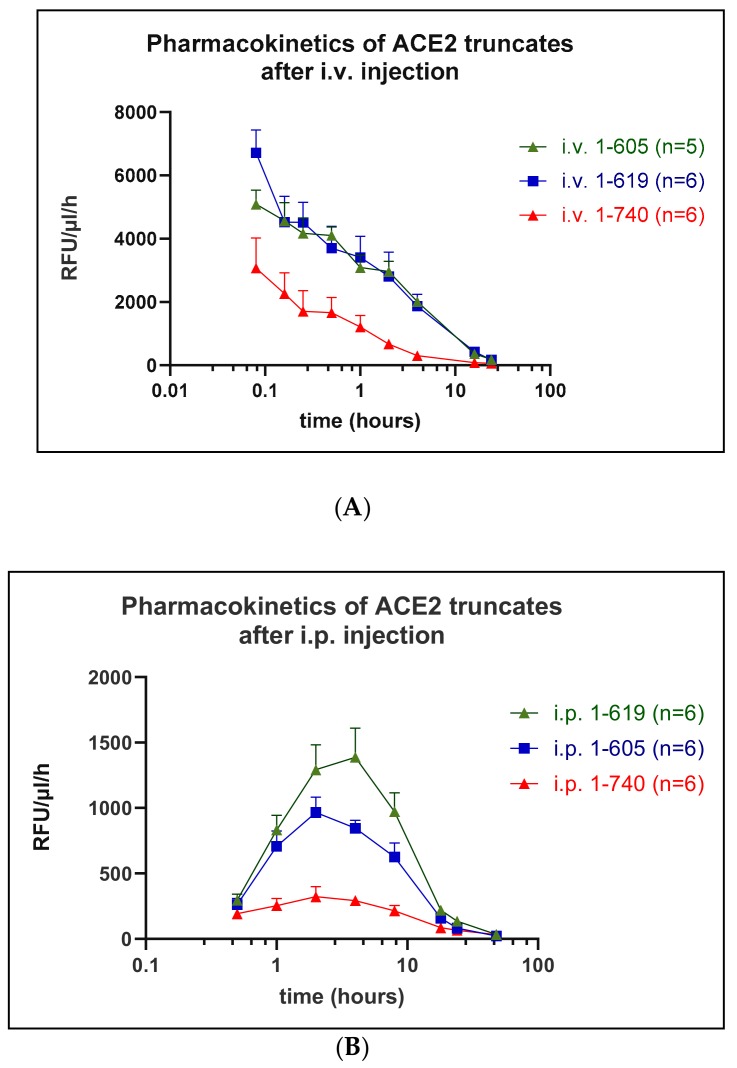
Pharmacokinetics of small ACE2 variants in vivo after i.v. (**A**) or i.p. injection (**B**). Wild-type mice (*n* = 5–6 per experiment) were injected with 1 µg/g BW of native murine rACE2 1-740 (reference protein) or mouse small rACE2 variants 1-619 and 1-605. Venous blood was collected at defined time points after bolus rACE2 injection and ACE2 activity measured using Mca-APK-Dnp fluorogenic substrate; RFU—relative fluorescence unit.

Pharmacokinetic profiles were determined in mice for rACE2 (1-619) and rACE2 (1-605) as compared to rACE2 (1-740) using the Mca-APK(Dnp) assay to measure ACE2 activity in serum collected in a time series. Following a single i.v. injection of each the rACE2 forms (1 µg/g BW) (see Methods), each had a relatively short distribution phase in the blood, followed by a longer elimination phase (Figure 6, Table 1). The distribution phase half-life of the 1-605 (58 ± 24 min) was not different from that of 1-619 (19 ± 5 min) and of native rACE2 (13 ± 2 min). Both 1-605 and 1-619 had a three times longer elimination phase half-life of 4.0 ± 0.5 (*p* = 0.054) and 4.2 ± 1.07 h (*p* = 0.029), respectively, compared to 1.35 ± 0.21 h for 1-740 (Table 1). The calculated area under the curve, which reflects the overall in vivo pharmacological efficacy, was also higher (around 4–5 times) for the two small rACE2 variants (*p* < 0.05 for both) as compared to native rACE2 1-740 (Table 1). In addition, the maximum serum activity of the two short ACE2 proteins was much higher than that of native rACE2 (Figure 6). The mean residence time was also higher for the rACE2 1-605 than for native rACE2 (*p* = 0.038), while for rACE2 1-619 variant the difference did not reach statistical significance (Table 1).

Following i.p. infusion, a method of delivery used in previous studies [6,7,15], as expected, there was a delay in achieving peak blood activity levels for both short rACE2s (1-619 and 1-605) and for rACE2-1-740 (Figure 6, Table 2) compared to levels obtained using i.v. administration (Table 1). Similar to the i.v. injections, after i.p. administration the maximum serum activity and the calculated AUC of native rACE2 1-740 was lower than that of the two shorter ACE2 variants (Figure 6). The elimination phase half-life and the mean residence time, however, were not lower compared to the two shorter ACE2 proteins (Table 2).

Overall, the small ACE2 variants 1-605 and 1-619 exhibited a higher serum activity than native rACE2 1-740 both after i.v and i.p. administration, and the 1-605 variants had the longest circulatory residence time after i.v injection (Table 1 and Table 2).

### 3.5. Demonstration of In Vivo Activity as Reflected by Blood Pressure-Lowering Effect of Short rACE2 Variants during Acute Ang II Infusion in Mice

**Figure 7:**
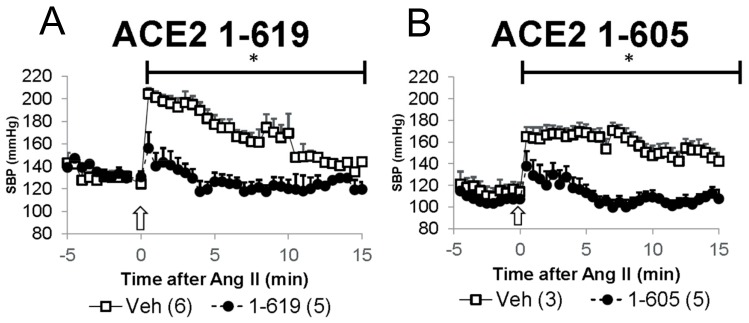
Pre-infusion of 1-619 (**A**) or 1-605 (**B**) rACE2 variants (filled circles) cause a faster recovery from acute Ang II-induced hypertension as compared to respective mice pre-infused with PBS as a vehicle (Veh; empty squares). *Y*-axis is SBP and *X*-axis indicates time (min) from Ang II bolus (arrow; 0.2 µg/g BW). Two-way ANOVA over time was used for comparisons of the experimental groups; * denotes *p* < 0.01.

The effect of short rACE2 on acute hypertension was examined in a model of angiotensin II-induced hypertension as previously described by us [6,7,15]. Mice were pretreated with either vehicle (PBS), rACE2 1-605 or rACE2 1-619, by a single i.p. injection at 1 h before receiving a bolus injection of Ang II (Figure 7). Blood pressure in mice was monitored continuously under light anesthesia in a time series at 30 s intervals that started five minutes before Ang II injection (see Methods). The injection of Ang II resulted in a rapid increase in systolic blood pressure (peak of 204 ± 5 and 169 ± 8 mm Hg) from a baseline (124 ± 9 and 114 ± 9 mm Hg) in the control groups that had received PBS as pre-injection vehicle (Figure 7A,B, respectively). This increase in SBP was efficiently mitigated to a similar extent in the both rACE2 treatment groups (Figure 7A,B).

### 3.6. Recovery of Urinary ACE2 Activity after Administration of Short rACE2 Variants to ACE2-Deficient Mice

Since normally ACE2 is present in the urine, to better assess whether there is any increase in urinary ACE2 activity after administration of short ACE2 variants, we injected them to ACE2 deficient (ACE2-/-) mice [26,27]. Urinary ACE2 activity was measured using the Mca–APK–Dnp fluorogenic substrate.

**Figure 8:**
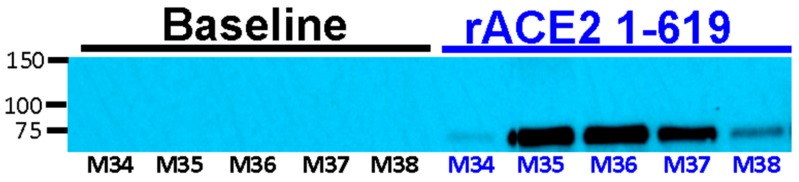
Western blot of urines collected before (Baseline) and after i.v. bolus of ACE2 1-619 variant (0–2 h) from five ACE2-deficient mice, showing an ACE2-immunoreactive band at the expected size of ~70 kD, consistent with the molecular size of the short rACE2 1-619 variant after, but not before, the injection. M34-M38 are ID numbers of the individual ACE2 deficient mice (*n* = 5) from which urines were obtained at baseline and within 2 h after mrACE2 1-619 infusion. Equal volumes (36 μL) of urines with similar creatinine concentrations (~10–20 mg/dL) were loaded per well.

As expected, no enzymatic ACE2 activity was detectable in baseline urines collected before infusion to ACE2-deficient mice ((−0.1 ± 0.2 RFU/µg creat/h, *n* = 5). The i.v. infusion of 1-619 and 1-605 resulted in increased ACE2 activity in mice (*n* = 5) to about the same extent (4.5 ± 1.8 and 5.1 ± 1.9, respectively). Urine was collected 1–2 h after the injection. Western blots, moreover, showed an ACE2-immunoreactive band after the rACE2 1-619 i.v. infusion (Figure 8).

### 3.7. Glomerular Filtration and Kidney Uptake of Small rACE2 Variants in ACE2-Deficient Mice

**Figure 9:**
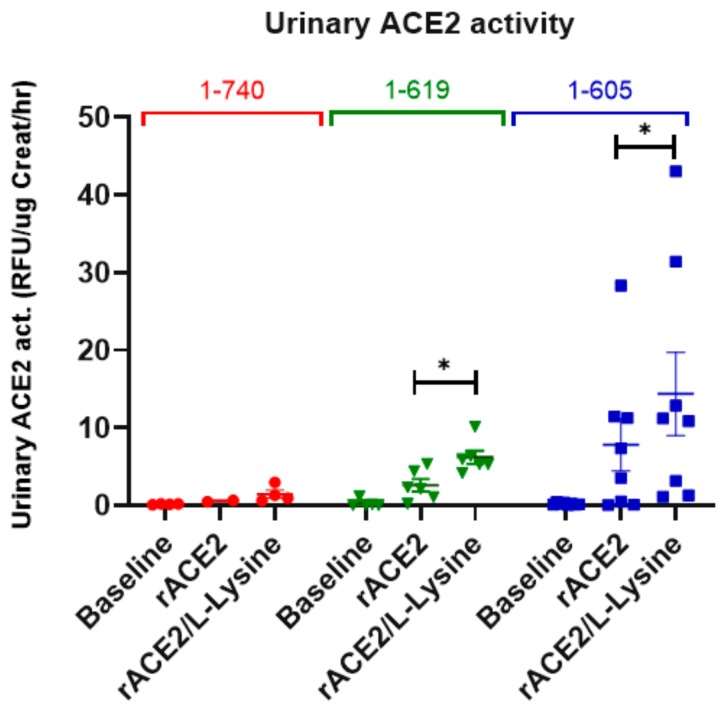
Glomerular filtration and tubular uptake of 1-605- (blue, *n* = 8) and 1-619-mACE2 (green, *n* = 6) as compared to native 1-740 rACE2 (red, *n* = 4) in ACE2-deficient mice. Immediately after voiding (baseline urine collection), ACE2-deficient mice were injected i.v. with 1 µg/g BW rACE2. Urine was collected again within the first 2 h after ACE2-injection (“rACE2”). L-Lysine was injected i.p. two hours after rACE2-injection and urine was collected within the next 2 h. (“rACE2/L-Lysine”). Recovery of ACE2-activity in the urine normalized to creatinine excretion is depicted. Repeated measures one-way ANOVA was used for comparisons within the experimental groups, followed by post-hoc analysis; * denotes *p* < 0.05 or *p* < 0.01.

To examine whether, after glomerular filtration, a tubular uptake of filtered rACE2s takes place, ACE2-deficient mice were infused with either rACE2-1-605, 1-619 or the native rACE2 1-740 as negative control. Subsequently, L-lysine (a blocker of proximal tubular protein reabsorption) was injected i.p. (Figure 9).

Before injection and after native rACE2 injection, urinary ACE2 activity was not detectable, consistent with the lack of glomerular filtration of the large native rACE2. The tubular reabsorption blocker, L-lysine, moreover, did not result in an appreciable gain in urinary ACE2 activity (Figure 9).

In contrast, both small recombinant variants (1-619 and 1-605) when infused to ACE2-deficient mice, resulted in a clear gain in urinary ACE2 activity. Blocking with L-lysine further increased urinary ACE2 activity, demonstrating that the two truncates are both filtered and reabsorbed by the tubules (Figure 9). In the aggregate, these data show that rACE2-605 and rACE2-619 must undergo glomerular filtration and subsequent proximal tubular reabsorption.

**Figure 10:**
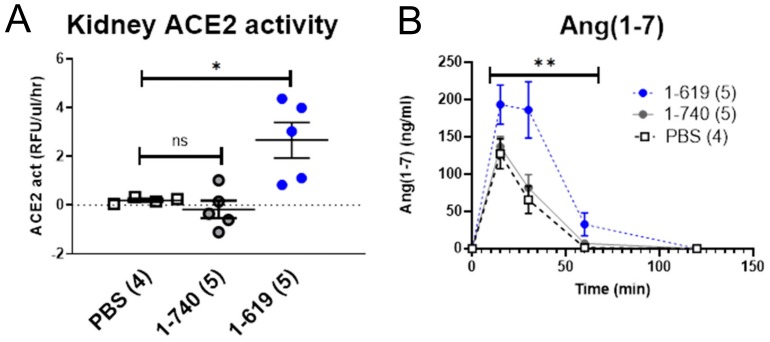
ACE2-deficient mice were infused i.v. with a bolus of PBS, native mouse rACE2 (1-740) or mouse rACE2 1-619 (1 µg/g BW). After 2 h, mice were perfused with saline and the kidneys removed. (**A**) A gain in kidney ACE2 activity was only appreciated in 1-619-infused mice. (**B**) In an ex vivo setting, kidney lysates (2 mg) were incubated with Ang II (10^−5^M) to assess Ang 1–7 formation over time by 2-way Anova. Higher Ang 1–7 formation was seen in 1-619 mice as compared to the two other groups; ** denotes *p* < 0.01 against 1-740 and *p* < 0.001 against PBS.

To examine whether kidneys from ACE2-deficient mice infused with ACE2 1-619 gain substantial ACE2 activity, the ACE2 619 truncate was injected and kidneys harvested after infusion (Figure 10). In isolated kidney cortices from ACE2-deficient mice, ACE2 activity was not detectable in either in PBS- or rACE2 1-740-infused mice. In contrast, kidney cortex ACE2 activity after rACE2 1-619 was clearly detectable (2.7 ± 0.7 RFU/µg prot./h) and significantly higher than in PBS (0.2 ± 0.1 RFU/µg prot./h, *p* < 0.05) and rACE2 1-740 groups (−0.2 ± 0.8 RFU/µg prot./h, *p* < 0.01) (Figure 10A). In ex vivo experiments, kidneys from 1-619-infused mice, when spiked with Ang II, also exhibited significantly higher Ang 1–7 formation than kidneys from PBS-mice (*p* < 0.001) or native rACE2- 1-740-infused mice (*p* < 0.01) by 2-way Anova (Figure 10B). This shows that kidney uptake translates into increased kidney ACE2 activity and capability of Ang II to Ang 1–7 conversion.

## 4. Discussion

In this study, we describe the design and testing of novel recombinant mouse ACE2 proteins of a molecular size (~69–71 kD) much shorter than the native ACE2. The two short ACE2 variants are very active systemically, but of a molecular size short enough to render them filterable through the kidney glomerular filtration barrier. These short variants, moreover, are re-absorbable by the kidney tubules and thus capable of amplifying kidney ACE2 activity and fostering the formation of Ang 1–7 from Ang II. This feature should make them attractive to combat a vast array of kidney diseases where the RAS is over-active, such as acute kidney injury.

We and others have previously identified two main ACE2-immunoreactive bands in mouse urine [12,18,19,20,21]. One band at about 110 kD corresponded to the molecular weight of native ACE2 and was likely a product of shedding, likely mediated by the metalloprotease ADAM17 [20,30,31] from the kidney apical tubular membrane, where ACE2 is abundantly expressed [12,31]. These observations prompted us to investigate if these shorter forms of about 75 kD are enzymatically active and, if so, to design shorter ones and test them for potential activity in vitro and in vivo. Here, we demonstrate that the natural ACE2 of 100–110 kDa can be degraded in the urine and kidney to shorter ACE2 fragments which retain ACE2 enzyme activity. That the 75 kD ACE2 band is a result of a proteolytic cleavage of the 110 kDa ACE2 was determined by demonstrating that a recombinantly generated native recombinant, ACE2 110 kDa, can be digested by kidney and urine proteases to a shorter band of similar size to the 75 kDa ACE2 fragment that was naturally observed in the urine (Figure 1 and Figure 2). In these experiments, we were also able to ascertain that the C-terminal end is the site, which is removed from native rACE2 without a loss in enzyme activity (Figure 3). This finding is consistent with crystallographic and homology studies for human ACE2 that indicated that on the C-terminal end there is a fairly large structural portion that has non-enzymatic function [11,32].

We next proceeded to demonstrate that longer exposure of the 110 kDa native rACE2 to proteases from kidneys resulted in the formation of even a shorter ~60–70 kDa band and showed that it still had very high ACE2 enzymatic activity (Figure 1). By contrast, in the urine, extended incubation of native rACE2 ultimately led to the disappearance of the 75 kDa ACE2 band and a loss of ACE2 activity. This suggests that, unlike the kidney, in which ACE2 truncates can prevail for an extended period of time, the urine is equipped with a repertoire of proteases that inactivate ACE2 by digesting it to small inactive fragments. We did not investigate, however, the specific proteases responsible for the cleavage of ACE2 or the cleavage site. Such studies should be done in the future.

Based on the findings of the ex vivo proteolysis of native rACE2, we then generated short ACE2 variants of varying lengths which were gradually truncated from the C-terminal end of ACE2. The shortest active variant was 605 AA long (~69 kDa) and all other longer variants exhibited enzymatic activity as well. In contrast, the short variant of 522 AA was enzymatically inactive (Figure 4). From this, our preliminary conclusion is that the minimum length requirement for ACE2 to be active has to be somewhere between 522 and 605 AA.

The two active short proteins 1-619 and 1-605 were then purified and their in vivo effects were compared with the native rACE2 and the half-lives of the injected recombinant proteins were evaluated (Figure 6; Table 1 and Table 2). Compared to the native rACE2′s blood half-life of 1.4 h after i.v. administration, ACE2 1-619 and 1-605 had a substantially extended half-life of around 4 h. In addition, the area under the curve for the short ACE2 proteins was about 4–5-fold higher than that of the native rACE2 after i.v. injection (Table 1) and 2–3-fold higher after i.p. administration (Table 2). It is also noteworthy that, with the same dose, the peak plasma ACE2 activity after the infusion of both short variants was substantially higher than that of native rACE2 (Figure 6), further suggesting a better pharmacological profile, as compared to the native rACE2 form. It should be noted, however, that this pharmacokinetic profile was based on ACE2 activity assessed against an artificial substrate. The catalytic efficiency towards the artificial substrate, likewise, was much higher than that of the native ACE2 (Figure 5A). In contrast, when the catalytic efficiency was assessed using the natural substrate, Ang II, then there were no significant differences between the short ACE2 variants and the native ACE2 (Figure 5B).

To test the systemic effect of the short ACE2 variants 1-605 and 1-619 in vivo, we used an established protocol for acute Ang II-induced hypertension [6,7]. Using this protocol previously, we have demonstrated that lowering plasma Ang II using native rACE2 effectively mitigated an increase in blood pressure caused by Ang II administration [7]. Similar to native ACE2 [6,7], both small ACE2 variants blunted the peak increase in blood pressure after Ang II infusion which normalized faster within five minutes or less. This shows that both small ACE2 variants efficiently degrade the excess of systemic circulating Ang II, thereby enhancing blood pressure recovery. Having demonstrated the systemic effect on blood pressure, we further studied whether both small ACE2 variants have any added effect to their systemic action at the local urinary and kidney level. ACE2 is normally present in the urine, and to better assess whether there is any increase in urinary ACE2 activity after administration of the short ACE2 variants, ACE2-deficient mice were used. In contrast to native rACE2 injection, both small recombinant variants resulted in a gain in urinary ACE2 activity. L-lysine, a tubular reabsorption blocker [28,29], further increased urinary ACE2 activity, suggesting that the two short ACE2 variants are both filtered and reabsorbed by the tubules. That the short ACE2 protein variant that is taken up by the kidney is indeed enzymatically active was shown by the presence of ACE2 activity in kidneys from ACE2 deficient mice that had been injected with ACE2 1-619. In addition, kidney cortex lysates from ACE2 1-619-injected mice were able to form significantly more Ang (1–7) from Ang II than kidney lysates from PBS- or native rACE2-injected mice. In the aggregate, these data show that mouse short ACE2 variants are active, and sufficiently small to be filtered by the kidney and, moreover, capable of increasing kidney ACE2 activity to the extent that Ang 1–7 formation from Ang II is increased (Figure 10). As a limitation of this study, we acknowledge that our studies were not performed in a blinded fashion and that a potential bias in the measurements of activity of ACE2 in the urine and at the kidney level were not prevented by stratified randomization of the treated animals. In this regard, the ACE2 knockout allowed us to demonstrate urine and kidney activity after injection of our short ACE2 variants relatively easily and show that this was not the case after the infusion of native ACE2. The use of native rACE2 as a control enhances the results obtained with the novel short ACE2 variants and is a strength of our design. Even though the pharmacokinetic data show the clear superiority of short rACE2 variants as compared to the native rACE2, we caution to emphasize that this effect may be dependent on the artificial substrate used to measure ACE2 activity.

Knowing that the kidney ACE2 activity can be increased by the administration of short ACE2 variants, the natural question is under what clinical conditions such kidney amplification could be useful. We speculate that disease conditions where the local kidney RAS is over-active would be the natural target. Several studies using diabetic models show activation of the RAS locally within the kidney by glucose, including an increase in Ang II production [33,34,35,36]. Additional evidence comes from findings of increased RAS components in the kidney and urines from rodent models of diabetic kidney disease (DKD) and in patients with DKD [16,37] and non-diabetic CKD [38,39]. There is a need for new approaches to counteract RAS overactivity that expand and improve on the existing ones which are based on blockade of Ang II formation or action. We have chosen ACE2 as a therapeutic target because this enzyme cleaves Ang II to form Ang (1–7) with the highest efficiency [6,26,40].

There are several potential indications of our small ACE2 variants for the treatment of kidney disease. In particular, we think that Acute Kidney Injury (AKI) may benefit from the administration of our small ACE2 variants as a way to calm down the kidney RAS, which is overactive in this clinical syndrome. The pathogenesis of AKI is complex, often involving hemodynamic and non-hemodynamic mechanisms [41,42,43,44]. Alterations in the RAS have been shown to contribute to the development of AKI and are associated with adverse outcomes both in experimental and clinical studies [45,46,47,48,49,50]. Several components of the kidney RAS, such as urine Angiotensinogen and Ang II, the main active peptide, are increased in experimental AKI caused by ischemia-reperfusion AKI [45,46,47,51,52,53,54,55,56] ACE inhibitors (ACEi) and angiotensin receptor antagonists (ARBs) may attenuate renal inflammation, injury and improve renal function in the ischemia-reperfusion model of AKI [57,58,59,60]. Intrarenal Ang II is reportedly elevated in AKI, which may worsen kidney tissue injury independently of its hemodynamic effect that helps maintain renal circulation [61,62,63]. Based on these considerations, we are currently studying the effect of ACE2 1-619 in a mouse model of AKI.

In summary, we have developed short ACE2 variants with improved pharmacokinetic properties, as compared to the native rACE2. The short ACE2 protein variants, unlike the intact ACE2 protein, undergo glomerular filtration and tubular uptake. The kidney uptake of enzymatically active short ACE2 variants can enhance the local formation of Ang (1–7) from Ang II. The administration of active small ACE2 variants offers therapeutic potential in the prevention and treatment of acute kidney injury by directly reducing kidney RAS overactivity while retaining their systemic effect on ACE2 activity amplification.

## Figures and Tables

**Table 1:** Mean ± SE pharmacokinetic parameters of native rACE2 1-740 and the small ACE2 variants 1-619 and 1-605 in blood circulation in mice after **i.v.** administration.

	ACE2 1-740 (*n* = 6)	ACE2 1-619 (*n* = 6)	ACE2 1-605 (*n* = 5)
Distribution Phase (t_1/2α_) (min)	13 ± 2	19 ± 5	58 ± 24
Elimination Phase (t_1/2β_) (h)	1.35 ± 0.21	4.2 ± 1.07 *	4.0 ± 0.5
AUC	6437 ± 1639	27,636 ± 6297 *	28,257 ± 1901 *
MRT (h)	0.86 ± 0.12	2.20 ± 0.8	3.30 ± 0.72 *

* denotes *p* < 0.05 or *p* < 0.01 vs. ACE2 1-740.

**Table 2:** Mean ± SE pharmacokinetic parameters of native rACE2 1-740 and ACE2 1-619 and 1-605 truncates in blood circulation in mice after **i.p**. administration.

	ACE2 1-740 (*n* = 6)	ACE2 1-619 (*n* = 6)	ACE2 1-605 (*n* = 6)
Distribution Phase (t_1/2α_) (min)	123 ± 47	70 ± 8	62 ± 9
Elimination Phase (t_1/2β_) (h)	8.68 ± 0.79	5.62 ± 0.32 *	6.77 ± 0.67
AUC	5145 ± 683	17,792 ± 2288 *	11,753 ± 1250 *
MRT (h)	15.0 ± 1.71	14.9 ± 1.16	12.9 ± 0.95

* denotes *p* < 0.05, *p* < 0.01 or *p* < 0.01 vs. ACE2 1-740.

## Supplementary Materials

The following are available online at https://www.mdpi.com/2218-273X/9/12/886/s1, Figure S1: Generation and Testing of recombinant ACE2 Protein Truncates; Figure S2: Purification of short mouse ACE2 1-619; Figure S3: Amino acid sequences and molecular weights of the mouse ACE2 truncates.
