# Supplementary material for: Novel Variants of Angiotensin Converting Enzyme-2 of Shorter Molecular Size to Target the Kidney Renin Angiotensin System
Source: Biomolecules. 2019 Dec 17;9(12):886. doi: 10.3390/biom9120886 (PMC6995632; doi:10.3390/biom9120886)
Supplement: Supplementary file 1 [file biomolecules-09-00886-s001.pdf]

SUPPLEMENTARY MATERIAL

Supplementary Material List:

Figure S1. Generation and Testing of recombinant ACE2 Protein Truncates

Figure S2. Purification of short mouse ACE2 1-619

Figure S3. Amino acid sequences and molecular weights of the mouse ACE2 truncates

Figure S1. Generation and Testing of recombinant ACE2 Protein Truncates.

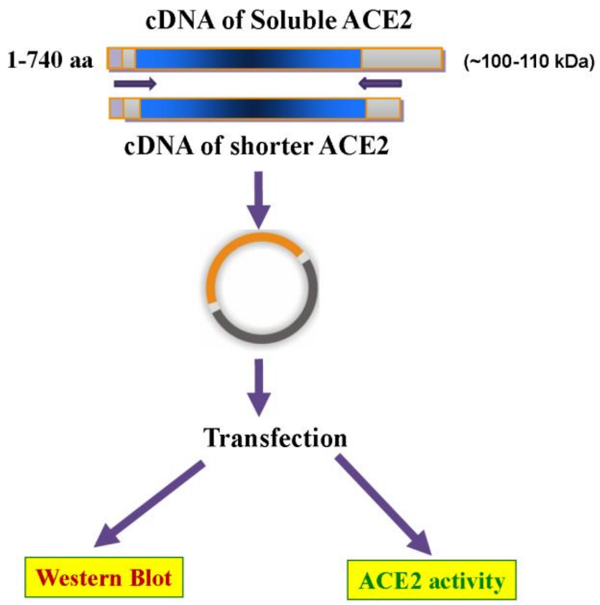

Supplementary Figure S1. Generation and Testing of recombinant ACE2 Protein Truncates.
A scheme showing a process of generation of a series of ACE2 truncates of varying lengths using cDNA of native mouse *ace2* 1-740AA as a template and *ace2*-sequence-specific primers. The generated C-terminally truncated *ace2* cDNAs were inserted into pcDNA plasmid and the constructs expressed in HEK293 cells. The conditioned culture media were used to verify the presence of the ACE2 protein by Western blot and its enzymatic functionality by measuring ACE2 activity using a fluorometric substrate Mca-APK-Dnp

Figure S2.

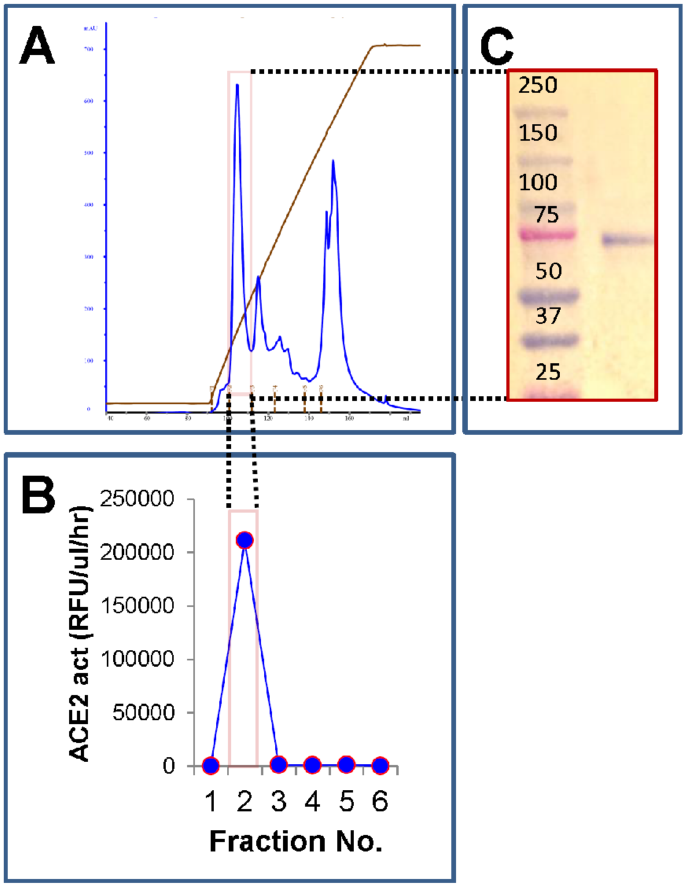

Supplementary Figure S2. Purification of short mouse ACE2 1-619. Conditioned serum-free medium from a clone of stably transfected HEK 293 cells that overexpress ACE2 1-619 was subjected to anion exchange Q-column. (**Panel A**). A chromatogram showing peaks of proteins eluted from the Q column (blue line) by applying increasing concentration of NaCl (brown line). Brown dashed lines perpendicular to x-axis indicate start points of collection of six elution fractions. The pink frame indicates a peak collected in fraction 2. Enzymatic assay (**Panel B**) using Mca-APK-Dnp substrate on all 6 fractions showing that ACE2 activity is restricted to fraction 2 (highlighted by pink frame) that represents the peak highlighted in panel A. This chromatographic step resulted in highly purified short ACE2 1-619 protein as shown on Brilliant Blue stained PVDF membrane (**Panel C**).

Figure S3.

A

1-522 AA (mouse ACE2; 522 AA long)

```
        10         20         30         40         50         60
MSSSSWLLLS LVAVTTAQSL TEENAKTFLN NFNQEAEDLS YQSSLASWNY NTNITEENAQ

        70         80         90        100        110        120
KMSEAAAKWS AFYEEQSKTA QSFSLQEIQT PIIKRQLQAL QQSGSSALSA DKNKQLNTIL

       130        140        150        160        170        180
NTMSTIYSTG KVCNPKNPQE CLLLEPGLDE IMATSTDYNS RLWAWEGWRA EVGKQLRPLY

       190        200        210        220        230        240
EEYVVLKNEM ARANNYNDYG DYWRGDYEAE GADGYNYNRN QLIEDVERTF AEIKPLYEHL

       250        260        270        280        290        300
HAYVRRKLMD TYPSYISPTG CLPAHLLGDM WGRFWTNLYP LTVPFAQKPN IDVTDAMMNQ

       310        320        330        340        350        360
GWDAERIFQE AEKFFVSVGL PHMTQGFWAN SMLTEPADGR KVVCHPTAWD LGHGDFRIKM

       370        380        390        400        410        420
CTKVTMDNFL TAHHEMGHIQ YDMAYARQPF LLRNGANEGF HEAVGEIMSL SAATPKHLKS

       430        440        450        460        470        480
IGLLPSDFQE DSETEINFLL KQALTIVGTL PFTYMLEKWR WMVFRGEIPK EQWMKKWWEM

       490        500        510        520
KREIVGVVEP LPHDETYCDP ASLFHVSNDY SFIRYYTRTI YQ
```

Theoretical Mw: 60,242 Da

B

1-605 AA (mouse ACE2; 605 AA long)

```
        10         20         30         40         50         60
MSSSSWLLLS LVAVTTAQSL TEENAKTFLN NFNQEAEDLS YQSSLASWNY NTNITEENAQ

        70         80         90        100        110        120
KMSEAAAKWS AFYEEQSKTA QSFSLQEIQT PIIKRQLQAL QQSGSSALSA DKNKQLNTIL

       130        140        150        160        170        180
NTMSTIYSTG KVCNPKNPQE CLLLEPGLDE IMATSTDYNS RLWAWEGWRA EVGKQLRPLY

       190        200        210        220        230        240
EEYVVLKNEM ARANNYNDYG DYWRGDYEAE GADGYNYNRN QLIEDVERTF AEIKPLYEHL

       250        260        270        280        290        300
HAYVRRKLMD TYPSYISPTG CLPAHLLGDM WGRFWTNLYP LTVPFAQKPN IDVTDAMMNQ

       310        320        330        340        350        360
GWDAERIFQE AEKFFVSVGL PHMTQGFWAN SMLTEPADGR KVVCHPTAWD LGHGDFRIKM

       370        380        390        400        410        420
CTKVTMDNFL TAHHEMGHIQ YDMAYARQPF LLRNGANEGF HEAVGEIMSL SAATPKHLKS

       430        440        450        460        470        480
IGLLPSDFQE DSETEINFLL KQALTIVGTL PFTYMLEKWR WMVFRGEIPK EQWMKKWWEM

       490        500        510        520        530        540
KREIVGVVEP LPHDETYCDP ASLFHVSNDY SFIRYYTRTI YQFQFQEALC QAAKYNGSLH

       550        560        570        580        590        600
KCDISNSTEA GQKLLKMLSL GNSEPWTKAL ENVVGARNMD VKPLLNYFQP LFDWLKEQNR

NSFVG
```

Theoretical Mw: 69,647 Da

C

1-619 AA (mouse ACE2; 619 AA long)

```
        10         20         30         40         50         60
MSSSSWLLLS LVAVTTAQSL TEENAKTFLN NFNQEAEDLS YQSSLASWNY NTNITEENAQ

        70         80         90        100        110        120
KMSEAAAKWS AFYEEQSKTA QSFSLQEIQT PIIKRQLQAL QQSGSSALSA DKNKQLNTIL

       130        140        150        160        170        180
NTMSTIYSTG KVCNPKNPQE CLLLEPGLDE IMATSTDYNS RLWAWEGWRA EVGKQLRPLY

       190        200        210        220        230        240
EEYVVLKNEM ARANNYNDYG DYWRGDYEAE GADGYNYNRN QLIEDVERTF AEIKPLYEHL

       250        260        270        280        290        300
HAYVRRKLMD TYPSYISPTG CLPAHLLGDM WGRFWTNLYP LTVPFAQKPN IDVTDAMMNQ

       310        320        330        340        350        360
GWDAERIFQE AEKFFVSVGL PHMTQGFWAN SMLTEPADGR KVVCHPTAWD LGHGDFRIKM

       370        380        390        400        410        420
CTKVTMDNFL TAHHEMGHIQ YDMAYARQPF LLRNGANEGF HEAVGEIMSL SAATPKHLKS

       430        440        450        460        470        480
IGLLPSDFQE DSETEINFLL KQALTIVGTL PFTYMLEKWR WMVFRGEIPK EQWMKKWWEM

       490        500        510        520        530        540
KREIVGVVEP LPHDETYCDP ASLFHVSNDY SFIRYYTRTI YQFQFQEALC QAAKYNGSLH

       550        560        570        580        590        600
KCDISNSTEA GQKLLKMLSL GNSEPWTKAL ENVVGARNMD VKPLLNYFQP LFDWLKEQNR

       610
NSFVGWNTEW SPYADQSIK
```

Theoretical Mw: 71,353 Da

Supplementary Figure S3. Amino acid sequences and the computed theoretical molecular weights of three mouse ACE2 protein truncates: 1-522 (A), 1-605 (B) and 1-619 (C) using the Expasy Informatics tool.
